# Transformer‐Based PET/CT Fusion Enables Preoperative Prediction and Prognostic Stratification of Spread Through Air Spaces in Lung Adenocarcinoma

**DOI:** 10.1002/advs.78076

**Published:** 2026-09-29

**Authors:** Xin‐Yu Zhu, Meng Meng, Zhen‐Zhen Wang, Deng‐Lu Lu, Ya‐Min Wei, Xu‐Chang Mi, Xing‐Yu Mu, Wei Fu

**Affiliations:** ^1^ Department of Nuclear Medicine The First Affiliated Hospital of Guilin Medical University Guilin China; ^2^ Laboratory Center Guangxi Key Laboratory of Metabolic Reprogramming and Intelligent Medical Engineering for Chronic Diseases The Second Affiliated Hospital of Guilin Medical University Guilin China; ^3^ Key Laboratory of Medical Biotechnology and Translational Medicine Education Department of Guangxi Zhuang Autonomous Region Guilin Medical University Guilin China; ^4^ Department of Nuclear Medicine Liuzhou Worker's Hospital Liuzhou China; ^5^ Department of Pathology The First Affiliated Hospital of Guilin Medical University Guilin China; ^6^ Department of Nuclear Medicine Nanxishan Hospital Guilin China

**Keywords:** deep learning, lung adenocarcinoma, multimodal fusion, PET/CT, prognostic biomarker, radiogenomics, spread through air spaces, transformer

## Abstract

Spread through air spaces (STAS) is associated with recurrence and unfavorable outcomes in lung adenocarcinoma, yet reliable preoperative identification remains challenging. This multicenter retrospective study includes 212 patients with 219 tumors across three institutions and develops a transformer‐based multimodal framework integrating eight complementary 2.5D PET and CT representations for preoperative STAS risk assessment. The model achieves an area under the receiver operating characteristic curve of 0.822 (95% CI, 0.725–0.920) in the independent external test cohort, with higher discrimination than the evaluated single‐modality and conventional fusion approaches. Beyond STAS classification, the continuous model‐derived risk score is associated with progression‐free survival after adjustment for pathological T stage, N stage, and maximum tumor diameter (HR, 4.24; 95% CI, 1.47–12.25; *P* = 0.008), although the limited number of progression events warrants cautious interpretation. Exploratory transcriptomic analyses across two public cohorts further nominate candidate programs involving cell adhesion, vesicle trafficking, complement activity, and metabolic remodeling. Together, these findings highlight the potential of multimodal PET/CT fusion to integrate morphological and metabolic information for noninvasive STAS risk stratification and support further prospective multicenter evaluation toward individualized surgical decision support.

## Graphical abstract

An eight‐stream 2.5D Transformer integrates complementary PET metabolic and CT morphologic information to estimate spread‐through‐air‐spaces risk before surgery in lung adenocarcinoma. The model generalizes across independent centers, while its continuous risk score independently stratifies progression‐free survival, supporting further prospective evaluation as an adjunct for individualized preoperative risk assessment and multidisciplinary surgical planning.

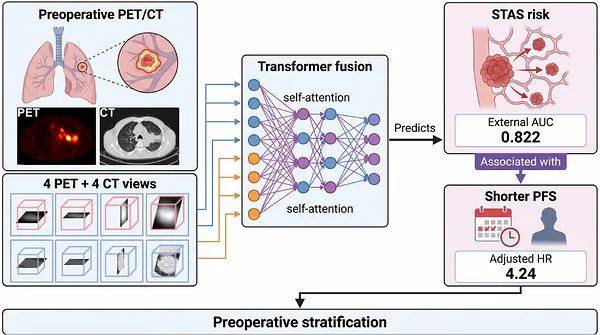

## 1 Introduction

Non‐small cell lung cancer (NSCLC) remains the leading cause of cancer‐related mortality worldwide, accounting for approximately 1.8 million deaths annually [1], with lung adenocarcinoma (LUAD) representing the most prevalent histological subtype and exhibiting a rising incidence trend globally [2]. Although surgical resection is the standard of care for LUAD [3], sublobar resection has gained prominence for its lung‐sparing advantages, demonstrating oncologic outcomes comparable to lobectomy in carefully selected patients [4]. Critically, the appropriateness of sublobar resection is contingent on pathological features that govern local recurrence risk, including spread through air spaces (STAS). STAS is a distinct pattern of invasion defined as micropapillary clusters, solid nests, or single tumor cells extending beyond the main tumor margin into adjacent alveolar spaces [5]. Accumulating evidence indicates that STAS is associated with locoregional recurrence and poor survival, particularly after sublobar resection [6, 7]. This creates a clinically important timing problem: the extent of resection must be selected before definitive STAS status is available from the resection specimen. Intraoperative frozen‐section assessment is not sufficiently sensitive or reproducible to reliably close this gap [8]. Consequently, a noninvasive preoperative STAS risk estimate could provide adjunctive information when balancing lobectomy against parenchyma‐sparing resection, while not replacing pathological diagnosis. Recent single‐cell and spatial transcriptomic work also links STAS with a distinct tumor microenvironment, including M2a macrophage enrichment, further supporting the biological heterogeneity of this invasive phenotype [9].

^18^F‐FDG PET/CT provides complementary morphological and metabolic information for characterizing tumor biology. In LUAD, CT depicts tumor morphology, growth patterns, and the tumor–lung interface, whereas PET provides information on metabolic activity and intratumoral heterogeneity. Emerging evidence suggests that STAS‐positive tumors may exhibit more aggressive imaging phenotypes, including ill‐defined margins and increased metabolic uptake; however, these features can be difficult to quantify by visual assessment alone [10, 11]. Recent PET/CT‐based radiomics and deep learning studies have further demonstrated the potential value of integrating metabolic and morphological imaging information for preoperative STAS prediction [12, 13]. Because neither modality alone fully captures the multidimensional imaging phenotype associated with STAS, effective integration of complementary PET and CT information may provide a more comprehensive basis for preoperative prediction.

Deep learning (DL) has shown strong potential in modeling complex, nonlinear relationships within medical imaging [14]. Unlike conventional radiomics, DL can preserve spatial context and inter‐slice dependencies, enabling a more comprehensive characterization of tumor morphology and biological heterogeneity. The Transformer architecture, through its multi‐head self‐attention mechanism, is well suited to modeling long‐range global dependencies among spatially distributed input tokens [15, 16]. This capability is particularly well‐suited to the multimodal STAS prediction task, where critical predictive information resides not only within individual PET or CT features but in their cross‐modal interactions—for example, the co‐occurrence of morphological interface irregularity (CT) and focal metabolic elevation (PET) may together constitute a stronger STAS signal than either feature independently. Conventional CNN‐based fusion approaches, which typically operate through local receptive fields or simple feature concatenation, may be limited in their ability to model such global cross‐modal relationships.

In this multicenter study, we pursued three interrelated objectives: (1) to develop and externally test a Transformer‐based 2.5D PET/CT fusion model for noninvasive preoperative STAS prediction in resectable LUAD; (2) to assess whether the model‐derived risk score provides prognostic information for PFS after adjustment for pathological T stage, N stage, and maximum tumor diameter; and (3) to explore, in a hypothesis‐generating manner, candidate transcriptomic programs associated with the imaging‐defined risk phenotype across two public cohorts.

## 2 Materials and Methods

### 2.1 Patients and Study Design

This retrospective study was approved by the Institutional Ethics Committee of the Affiliated Hospital of Guilin Medical University (Approval No.: 2025IITLL‐60), with the requirement for informed consent waived. A total of 315 consecutive patients with primary LUAD were initially recruited from three medical institutions between May 2020 and December 2024. Inclusion criteria were as follows: (1) preoperative ^18^F‐FDG PET/CT performed within two weeks before surgery without intervening antitumor therapy; (2) primary LUAD confirmed by postoperative pathological examination; and (3) availability of complete clinical, imaging, and follow‐up data. Exclusion criteria were: (1) synchronous multiple primary lung cancers within the same lobe; (2) poor PET/CT image quality precluding reliable segmentation; (3) receipt of antitumor therapy before the index PET/CT examination; and (4) pure ground‐glass nodule (GGN) morphology on CT without any solid component, as STAS is not assessable in this subgroup.

The TCGA‐LUAD and GEO‐NSCLC datasets from The Cancer Imaging Archive (TCIA) were used for exploratory radiogenomic analysis [17, 18]. Of the TCGA‐LUAD patients with available PET/CT imaging, 14 had matched RNA sequencing data and were used exclusively for radiogenomic hypothesis exploration; this cohort was not used for primary model performance validation due to the limited sample size. Similarly, 68 GEO‐NSCLC patients with available PET/CT imaging and matched RNA‐seq data were used for cross‐cohort transcriptomic comparison. These analyses are explicitly designated as exploratory. Exclusion criteria for TCIA patients were: (a) poor PET/CT image quality; and (b) absence of RNA‐seq data.

The final enrolled cohort comprised 212 patients with 219 tumors: 134 patients from The First Affiliated Hospital of Guilin Medical University (Center 1), 29 from Guilin Nanxishan Hospital (Center 2), and 49 from Liuzhou Workers' Hospital (Center 3). Center 1 contributed the training cohort (107 patients/tumors) and the internal validation cohort (27 patients/tumors), whereas Centers 2 and 3 together constituted the external test cohort (78 patients with 85 tumors). STAS classification analyses were performed at the tumor level, whereas survival analyses were performed at the patient level. The internal validation cohort (*n* = 27) is acknowledged to be underpowered for independent model validation; its findings should therefore be interpreted as indicative rather than confirmatory.

### 2.2 Clinical and Pathological Data Collection

Clinical and pathological data, including age, sex, tumor location, surgical approach, and maximum tumor diameter, were extracted from medical records. Tumor staging was performed according to the 8th edition of the AJCC/UICC staging system. PET/CT‐derived quantitative parameters—including maximum standardized uptake value (SUV_max_), mean standardized uptake value (SUV_mean_), metabolic tumor volume (MTV), and total lesion glycolysis (TLG)—were obtained from preoperative imaging. Details regarding the construction of the clinical model are provided in Note S1.

The TCGA‐LUAD and GEO‐NSCLC cohorts provided imaging and clinical data from patients with publicly available records. STAS status for tumors from Centers 1–3 was obtained from postoperative pathology reports. For the TCGA‐LUAD cohort, STAS status was re‐evaluated by pathologists from Center 1 in pulmonary pathology (Y.W., with more than 5 years of experience).

The primary clinical endpoint of this study was progression‐free survival (PFS), defined as the time (in months) from the date of surgery to radiologically confirmed recurrence or the last follow‐up without progression. Recurrence was defined as any local, regional, or distant disease progression identified based on CT and/or PET/CT findings. Standard follow‐up included chest CT at 3‐month intervals for the first 2 years, and 6‐month intervals thereafter, with ^1^ ^8^F‐FDG PET/CT performed at the discretion of the treating physician when recurrence was suspected. Biopsy of recurrent lesions was not routinely required and was performed only when imaging findings were equivocal or when histological confirmation was necessary for therapeutic decision‐making.

### 2.3 Image Acquisition and Tumor Segmentation

^18^F‐FDG PET/CT scans were obtained at the three clinical institutions using different PET/CT systems (Philips Ingenuity TF at Center 1, GE Discovery MI at Center 2, and Siemens BIOGRAPH mCT S(20)‐3R at Center 3). To reduce technical heterogeneity across scanners and acquisition protocols, a common modality‐specific preprocessing pipeline was applied before feature extraction. PET images were converted to body‐weight‐normalized standardized uptake values and resampled to 3 × 3× 3 mm^3^ isotropic voxels using linear interpolation; CT images were restricted to a lung‐window intensity range of ‐1000 to 300 HU and resampled to 1 × 1 × 1 mm^3^. Fixed discretization settings were then applied as detailed in Note S2.1. The same preprocessing rules were used across centers; no center‐specific feature transformation was introduced. Three‐dimensional tumor regions of interest (ROIs) were manually delineated on PET and CT images using 3D‐Slicer software (v5.6.2) by two nuclear medicine physicians (Z.Z.W. and X.Y.M., 8 years of experience), who were blinded to pathological outcomes. Inter‐observer reproducibility was assessed in 30 randomly selected tumors independently segmented by both physicians (ICC: PET 0.88; CT 0.82). A total of 4032 radiomic features were extracted using PyRadiomics for the conventional radiomics comparator (Note S2.2 and Figure S2).

To capture tumor heterogeneity from multiple spatial dimensions while balancing computational efficiency, four types of 2.5D image modules were constructed for both PET and CT: Multi‐Planar Imaging (MPI), Multi‐Slice Imaging (MSI), Multicenter Imaging (MCI), and Multi‐Angle Imaging (MAI) (Figure 1; Note S2.3). For each module, three slices were stacked along the channel axis and resized to 256 × 256 pixels, serving as the standardized input for the deep learning model. This resulted in eight parallel input streams (4 PET + 4 CT).

**FIGURE 1:**
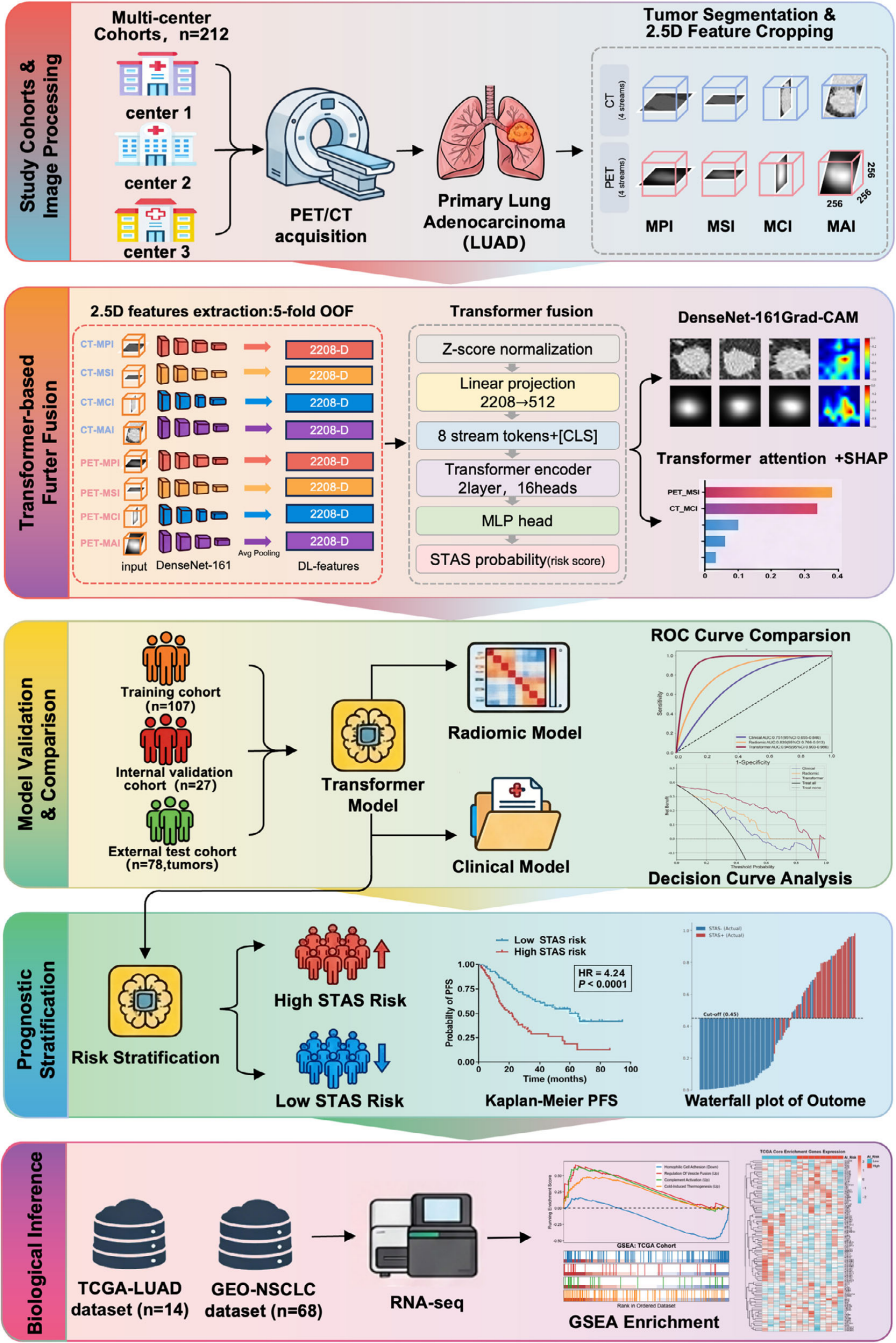
Workflow for tumor segmentation, deep learning feature extraction, Transformer‐based model construction, and downstream analysis. Four 2.5D representations were generated for each PET and CT modality, resulting in eight parallel input streams. Each DenseNet161 branch generated a 2,208‐dimensional feature vector, which was projected to 512 dimensions before Transformer‐based multimodal fusion.

### 2.4 2.5D Model Construction and Feature Extraction

The DenseNet161 architecture was adopted as the backbone network. Eight parallel image streams were independently trained on the training cohort. The model was initialized with ImageNet‐pretrained weights and optimized using the Stochastic Gradient Descent (SGD) optimizer with an initial learning rate of 0.01. A fixed batch size of 32 and a maximum of 100 epochs were used. Regularization techniques included dropout with a rate of 0.2 and L2 weight decay of 1e‐4. Data augmentation (random flipping and cropping) and early stopping were employed to prevent overfitting. Early stopping monitored loss on the internal validation set with a patience of 10 epochs.

For feature generation in the training cohort, a stratified fivefold out‐of‐fold (OOF) strategy was used so that each tumor's downstream Transformer fusion features were obtained from a DenseNet161 model that had not been fitted using that tumor's label. For each 2.5D stream, the fivefold‐specific DenseNet161 models were subsequently evaluated in the internal validation cohort, and the model achieving the highest AUC was selected and fixed as the branch‐specific inference model. This selected model was used to extract features from both the internal validation and external test cohorts. The external test cohort was not involved in model training, model selection, early stopping, or hyperparameter tuning. Detailed procedures are provided in Note S3.3.

After branch training, 2,208‐dimensional feature vectors were extracted from the final DenseNet161 dense block (global average pooling) for each PET or CT stream and used for subsequent Transformer fusion. Grad‐CAM was used only as a qualitative branch‐level spatial attribution analysis. For each independently trained DenseNet161 branch, the model was fixed in evaluation mode, and gradients were computed with respect to the STAS‐positive logit using denseblock.denselayer24.conv2 at the final DenseNet161 convolutional layer. The displayed Grad‐CAM maps are direct post hoc transformations of individual DenseNet branch outputs and do not directly visualize the final Transformer fusion decision. Further implementation is provided in Note S3.

### 2.5 Transformer‐based Multimodal Fusion

Following the completion of DenseNet161 backbone training and out‐of‐fold feature extraction, the resulting 2,208‐dimensional feature vectors from all eight 2.5D streams constituted the input to the Transformer‐based multimodal fusion stage. This two‐stage pipeline separates branch‐level spatial feature extraction (Stage 1: DenseNet161) from downstream cross‐modal interaction modeling (Stage 2: Transformer encoder), allowing the DenseNet‐derived representations to be subsequently integrated through Transformer‐based multimodal fusion. To model cross‐modal interactions among the PET and CT streams, the 2,208‐dimensional feature vectors were projected into a 512‐dimensional latent space before Transformer encoding. The core architecture consisted of a two‐layer Transformer encoder with 16‐head multi‐head self‐attention. This configuration allowed the model to concurrently attend to diverse spatial and metabolic correlations across different PET/CT views. A learnable class token was prepended to the feature sequence to aggregate holistic multimodal information. The final state of the encoder was processed by a Multilayer Perceptron (MLP) classification head comprising layer normalization and a direct linear projection, culminating in a two‐node softmax layer for STAS prediction. Attention weights were summarized as cohort‐level mean ± SEM to describe relative model focus across streams (Note S4). SHAP was used as a complementary post hoc attribution analysis to characterize the direction and magnitude of stream‐level contributions to the STAS‐positive model output relative to a reference input. Specifically, Kernel SHAP was applied at the level of the eight PET/CT feature streams, with each 2,208‐dimensional stream treated as a single grouped feature. These attribution analyses were considered descriptive rather than causal measures of feature importance. Ablation experiments compared Transformer fusion with early fusion, late fusion, and a reduced single‐view Transformer strategy (Note S6). Hyperparameters for both model stages were selected empirically during model development based on training convergence and internal‐validation performance; the external test cohort was not used for hyperparameter tuning or model selection.

### 2.6 Prognostic Stratification

To investigate the prognostic value of STAS in relation to PFS, the continuous model output was designated as the STAS risk score. The optimal threshold was determined via the Youden index in the training set. Survival analyses were performed at the patient level. For patients with multiple tumors, the highest model‐derived STAS risk score was used to represent the patient's risk phenotype. Kaplan–Meier curves and log‐rank tests were used for survival comparison. To assess the independent prognostic value of the STAS risk score for PFS, a multivariate Cox proportional hazards model was constructed incorporating the following covariates: STAS risk score (continuous), pathological T‐stage (T1 vs. T2/T3), N‐stage (N0 vs. N+), and maximum tumor diameter (continuous). The proportional hazards assumption was verified using Schoenfeld residuals. The dichotomized risk groups are intended for cohort‐level stratification in this study; the cutoff has not been prospectively calibrated as a stand‐alone surgical decision threshold.

To assess the robustness of the prognostic association across clinically relevant strata, an exploratory subgroup analysis was additionally performed in the pooled cohort according to age (≤ 60 vs. > 60 years), sex, pathological T stage (T1 vs. T2‐T3), N stage (N0 vs. N+), PET SUV_max_ (≤ 4.2 vs. > 4.2), and maximum tumor diameter (≤ 20 vs. > 20 mm). These subgroups were selected post hoc based on routine clinical availability and potential relevance to recurrence risk. Within each stratum, hazard ratios (HRs) for PFS comparing the transformer‐defined high‐ and low‐risk groups were estimated using Cox proportional hazards models, and interaction terms were used to assess effect heterogeneity. Analyses were performed using the “survival” and “forestploter” packages in R (version 4.2.0).

### 2.7 Exploratory Radiogenomic Analysis

For exploratory biological analysis, the pre‐trained Transformer model was applied to the TCGA‐LUAD and GEO‐NSCLC cohorts in an inference‐only manner to generate STAS risk scores, with model parameters fixed and no retraining performed. Patients were stratified into high‐ and low‐STAS‐risk groups using the training cohort‐derived cutoff. The TCGA cohort was stratified into high‐risk (*n* = 8) and low‐risk (*n* = 6) groups; the GEO cohort was stratified into high‐risk (*n* = 22) and low‐risk (*n* = 46) groups. These public‐cohort analyses were not used as confirmatory STAS validation because of the small matched TCGA sample and the absence of pathological STAS labels in GEO.

Transcriptomic profiles from both cohorts underwent independent Gene Set Enrichment Analysis (GSEA) referencing the Gene Ontology Biological Process (GOBP) collections [19]. Genes were pre‐ranked by DESeq2‐derived Wald statistics, and enrichment was executed with 1,000 permutations using the clusterProfiler R package [20, 21]. To identify cross‐cohort concordant pathways, a directional Venn intersection analysis was performed: pathways that were statistically significant (FDR‐adjusted *q* < 0.05) and showed concordant directional enrichment (showed the same direction of enrichment based on the sign of the Normalized Enrichment Scores (NES)) in both cohorts were retained as candidate pathways. NES of these concordantly enriched pathways were compared descriptively between the two cohorts to assess directional agreement. Leading‐edge genes within each consensus pathway were visualized by heatmap (ComplexHeatmap R package), and pathway enrichment plots were generated using the gseaVis package.

### 2.8 Statistical Analysis

Statistical analyses were performed using Python (version 3.7) and R software (version 4.2.0). Continuous variables were compared using Student's *t*‐test or Mann–Whitney *U* test for two‐group comparisons and one‐way ANOVA or Kruskal–Wallis test for three‐group comparisons, as appropriate. Categorical variables were compared using the chi‐square test or Fisher's exact test. STAS classification analyses were tumor‐level; for multivariable logistic regression, generalized estimating equations (GEE) were used to account for within‐patient correlation among patients with multiple tumors. Survival analyses were patient‐level. The risk‐score threshold was derived by the Youden index in the training cohort only. Multivariable Cox regression included four covariates and 37 progression events, corresponding to approximately 9.25 events per variable; therefore, hazard‐ratio precision was interpreted cautiously. The proportional hazards assumption was evaluated with Schoenfeld residuals, and model discrimination was summarized with Harrell's C‐index. PFS was compared using Kaplan–Meier curves and log‐rank tests. All tests were two‐sided; *P* < 0.05 was considered statistically significant.

## 3 Results

### 3.1 Baseline Cohort Characteristics and Analytical Units

The study included 107 patients in the training cohort, 27 patients in the internal validation cohort, and 78 patients in the external test cohort (Figure S1). The recurrence rate was 17.4% with a median follow‐up of 21.6 months. Baseline characteristics are summarized in Table S1. For STAS classification, these cohorts comprised 107, 27, and 85 tumors, respectively. In univariable comparisons, STAS‐positive tumors showed significantly higher conventional PET metabolic parameters, including SUV_max_, SUV_mean_, SUV_peak_, and 40% TLG (all *P* < 0.05, Table 1). However, the standalone clinical model based on conventional quantitative parameters showed only moderate discrimination, supporting the added value of multimodal deep learning. Results from the conventional radiomics and clinical models are presented in Note S5 and Table S2. For clarity, the reported cohort sizes for STAS classification refer to tumors: 107 training tumors, 27 internal‐validation tumors, and 85 external‐test tumors. The external test cohort included 78 patients with 85 tumors. Survival analyses used 212 patient‐level records.

**TABLE 1:** Characteristics of tumors between STAS‐positive and STAS‐negative groups in all cohorts.

	Training cohort (*n* = 107)			Internal validation cohort (*n* = 27)			External test cohort(*n* = 85)		
Characteristics	STAS Negative (*n* = 66)	STAS Positive (*n* = 41)	*P* value^*^	STAS Negative (*n* = 18)	STAS Positive (*n* = 9)	*P* value^*^	STAS Negative (*n* = 58)	STAS Positive (*n* = 27)	*P* value^*^
Age (years)	60.09 ± 9.68	60.66 ± 8.80	0.644	61.61 ± 11.89	61.00 ± 11.89	0.896	61.73 ± 6.33	61.70 ± 10.69	0.852
Sex			0.120			0.077			0.002
Female	42(63.6)	19(46.3)		12(66.7)	2(22.2)		47(81.1)	12(44.4)	
Male	24(36.4)	22(53.7)		6(33.3)	7(77.8)		11(18.9)	15(55.6)	
Maximum tumor diameter (mm)	22.33 ± 11.44	24.46 ± 10.05	0.142	20.17 ± 9.59	23.22 ± 9.51	0.410	18.61 ± 7.75	23.11 ± 10.69	0.053
SUV_max_	4.33 ± 5.24	7.25 ± 4.10	0.001	4.12 ± 3.85	11.58 ± 5.34	0.001	4.28 ± 3.15	8.17 ± 5.62	0.001
SUV_mean_	2.62 ± 3.21	4.42 ± 2.50	0.001	2.49 ± 2.40	7.05 ± 3.35	0.001	2.61 ± 1.94	5.00 ± 3.30	0.001
SUV_peak_	3.16 ± 3.78	5.42 ± 3.35	0.001	3.00 ± 2.84	8.01 ± 3.85	0.003	3.06 ± 1.76	5.14 ± 3.46	0.009
40%_TLG	21.56 ± 52.62	53.06 ± 146.36	0.001	21.11 ± 55.46	28.79 ± 19.73	0.013	8.62 ± 13.23	30.56 ± 68.65	0.001
T			0.792			1			0.221
T1	50(75.8)	33(80.5)		15(83.3)	8(88.9)		53(91.4)	22(81.5)	
T2	13(19.7)	7(17.1)		3(16.7)	1(11.1)		5(8.6)	4(14.8)	
T3	3(4.5)	1(2.4)		0	0		0	1(3.7)	
N			0.002			0.044			0.001
N0	56(84.8)	28(68.3)		16(89.0)	5(55.6)		56(96.6)	15(55.6)	
N1	10(15.2)	6(14.6)		1(5.5)	4(44.4)		1(1.7)	5(18.5)	
N2	0	7(17.1)		1(5.5)	0		1(1.7)	7(25.9)	
M			1.000			1.000			1
M0	66(100.0)	41(100.0)		18(100.0)	9(100.0)		58(100.0)	27(100.0)	

Data are presented as n (%) for categorical variables and mean ± standard deviation (SD) for continuous variables.

*P values were calculated using the Student's *t*‐test for continuous variables and the chi‐square test or Fisher's exact test for categorical variables, as appropriate.

### 3.2 Single‐Modality Deep Learning Model Evaluation

In the external test cohort, all single‐modality 2.5D branches demonstrated moderate discrimination, with AUC values ranging from 0.584 (PET_MPI) to 0.743 (CT_MSI; Table S3). CT‐based branches consistently outperformed their PET counterparts (CT AUC range: 0.663–0.743 vs. PET AUC range: 0.584–0.688), suggesting that morphological features captured by CT contribute more discriminatively to STAS classification than metabolic features from PET in single‐modality configurations. MSI and MPI streams generally outperformed MCI and MAI streams, indicating that sequential slice information and maximal cross‐sectional coverage are the most informative 2.5D representations for STAS characterization.

### 3.3 Transformer‐Based Multimodal Fusion Performance

The Transformer fusion model substantially outperformed all single‐modality branches (Figure 2A). It achieved an AUC of 0.945 (95% CI: 0.903–0.986) in the training cohort, 0.824 (95% CI: 0.660–0.987) in the small internal validation cohort, and 0.822 (95% CI: 0.725–0.920) in the external test cohort (Figure 2B; Figure S3A,D). Given the limited size of the internal validation cohort (*n* = 27), its performance estimate should be interpreted as supportive rather than confirmatory; the external test cohort served as the principal independent assessment of model generalizability. In the TCGA cohort, the AUC was 0.796 (95% CI: 0.505–1.000), but the extremely small sample (*n* = 14) and wide confidence interval preclude confirmatory interpretation. In the external test cohort, the Transformer fusion model achieved a higher AUC (0.822) than early fusion (0.706), late fusion (0.746), and Transformer_MCI (0.762) (Figure 2C; Table S4), suggesting a performance advantage of the proposed fusion strategy in this dataset. These results support an incremental performance advantage of the self‐attention mechanism in this dataset, while the training‐to‐external AUC decrease indicates residual overfitting. Decision‐curve and calibration analyses are shown in Figure 2D,F and Figure S3; their clinical utility requires prospective confirmation. Figure 2E further illustrates the relationship between the Transformer‐derived risk ranking, pathological STAS status, and model‐derived feature patterns at the tumor level.

**FIGURE 2:**
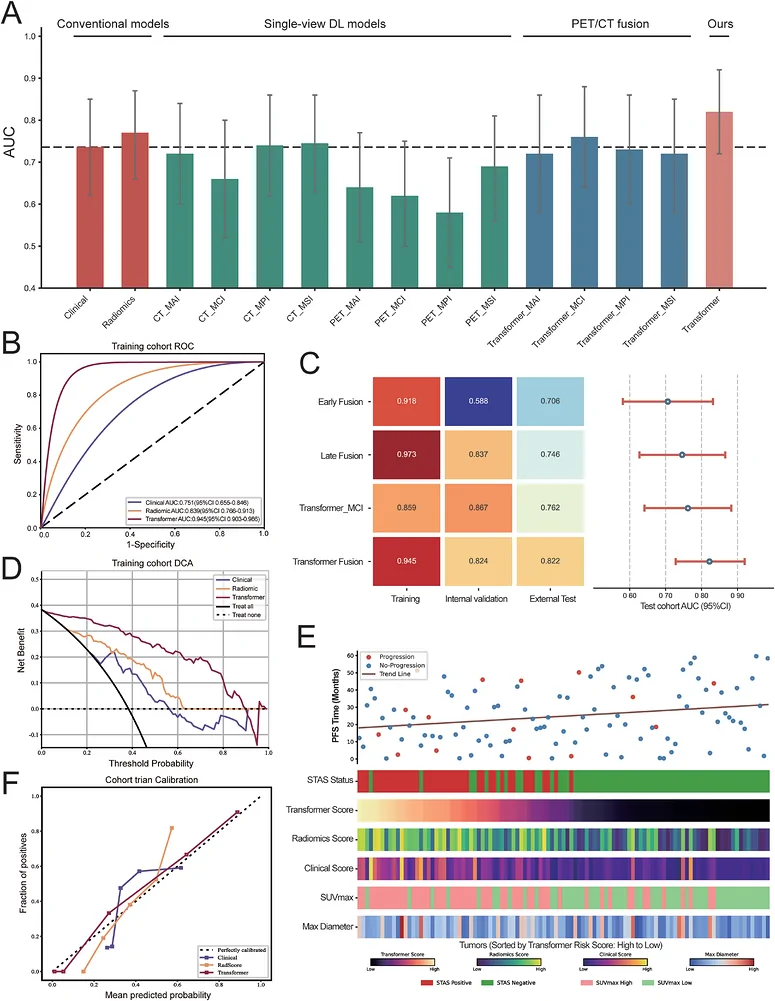
Performance evaluation and clinical utility comparison of the Transformer‐based model, radiomics model, and clinical model. (A) Bar chart comparing AUC values across model families. (B) ROC curve of the Transformer fusion model in the training cohort. (C) ROC curves comparing Transformer fusion with alternative fusion strategies in the independent external test cohort; pairwise DeLong *P* values are shown. (D) Decision‐curve analysis in the training cohort. (E) Heatmap of tumor‐level STAS outcomes and model‐derived feature contributions, with tumors sorted by Transformer risk score; columns represent individual tumors. (F) Calibration curve comparing predicted and observed STAS probabilities in the training cohort.

### 3.4 Model Interpretability and Feature Visualization

Given the high‐dimensional nature of the input data, for each case, the eight 2.5D PET/CT streams were represented by eight 2,208‐dimensional feature vectors, with each stream representation serving as one input token to the Transformer. To characterize the attention patterns of the Transformer‐based framework (Figure 3A), we quantitatively extracted the attention weights from the Transformer encoder. Global weight analysis (Figure 3B) showed relatively higher mean attention weights for PET_MSI (0.20) and CT_MCI (0.18). Notably, the remaining six feature sequences exhibited weights ranging from approximately 0.08–0.12. The distributed attention pattern is consistent with the model drawing information from multiple 2.5D perspectives rather than concentrating exclusively on a single stream. The attention weights reflect the average cohort‐level pattern of relative focus across the eight streams and should be interpreted descriptively rather than as definitive measures of stream‐specific importance. Individual‐level attribution variability and stability across retraining or perturbation were not formally quantified; therefore, the attention analysis is presented as an exploratory description of model behavior.

**FIGURE 3:**
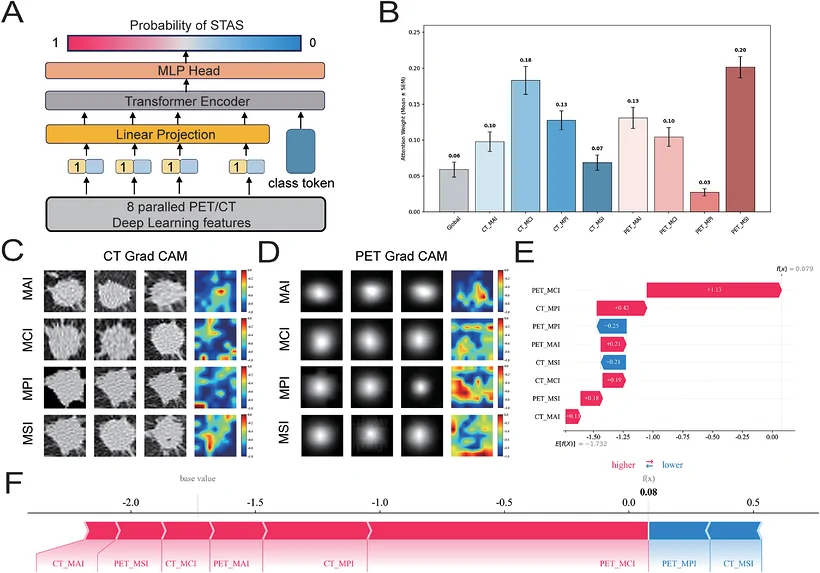
Descriptive interpretability analyses of the multimodal framework. (A) Transformer architecture. (B) Training‐cohort mean attention weights ± SEM across the eight streams; these weights describe relative model focus and are not causal importance measures. (C,D) Branch‐level Grad‐CAM visualizations generated from denseblock.denselayer24.conv2 of independently trained DenseNet161 branches. (E) SHAP force plot for a representative STAS‐positive case from the internal validation cohort. (F) Corresponding grouped stream‐level SHAP contributions relative to the training‐cohort‐derived reference representation.

As illustrated in the representative STAS‐positive case (Figure 3C–F), Grad‐CAM heatmaps (Figure 3C,D) qualitatively highlighted tumor‐associated regions, including portions of the tumor periphery and tumor–lung interface. Because these maps were generated from individual DenseNet branches, they represent branch‐level spatial attribution rather than a direct explanation of the final Transformer fusion decision. To characterize stream‐level contributions for an individual case, grouped Kernel SHAP analysis was performed (Figure 3E,F) that an STAS‐positive model logit of *f(x)* = 0.08, with PET_MCI (+1.13) and CT_MPI (+0.42) providing the largest positive stream‐level contributions relative to the reference input. These visualization analyses provide descriptive and hypothesis‐generating information about model behavior. In contrast, Figure S4 presents the corresponding SHAP analysis for a representative STAS‐negative patient, in whom the model generated a markedly low‐risk prediction (*f(x)* = −2.535 below base value), with PET_MSI emerging as the largest negative stream‐level contribution (−1.34) in this case. The opposing attribution patterns in these representative cases illustrate model behavior but do not establish attribution stability or biological mechanism.

### 3.5 Prognostic Stratification

Patients with increasing Transformer risk scores exhibited progressively higher rates of pathologically confirmed STAS (Figure 4A,B; *P* < 0.001). Similar cohort‐specific patterns were observed in the internal validation and external test cohorts (Figure S5A–C and F–H). The STAS‐positive group had significantly higher risk scores than the STAS‐negative group (Figure 4C, *P* < 0.001). Survival analysis showed that the STAS risk score was associated with PFS [22]. In pooled survival analysis, high‐risk patients exhibited significantly shorter PFS (HR = 3.55, 95% CI: 1.76–7.14, log‐rank *P* < 0.0001; Figure 4D). Cohort‐specific separation was observed in the training cohort (*P* = 0.004) and external test cohort (*P* = 0.019), whereas the small internal validation cohort did not reach statistical significance (*P* = 0.108) and should not be considered confirmatory (Figure 4E–G). A sensitivity analysis excluding patients with less than 6 months of follow‐up showed a similar pattern of survival separation (Figure S6), although the limited number of progression events warrants cautious interpretation.

**FIGURE 4:**
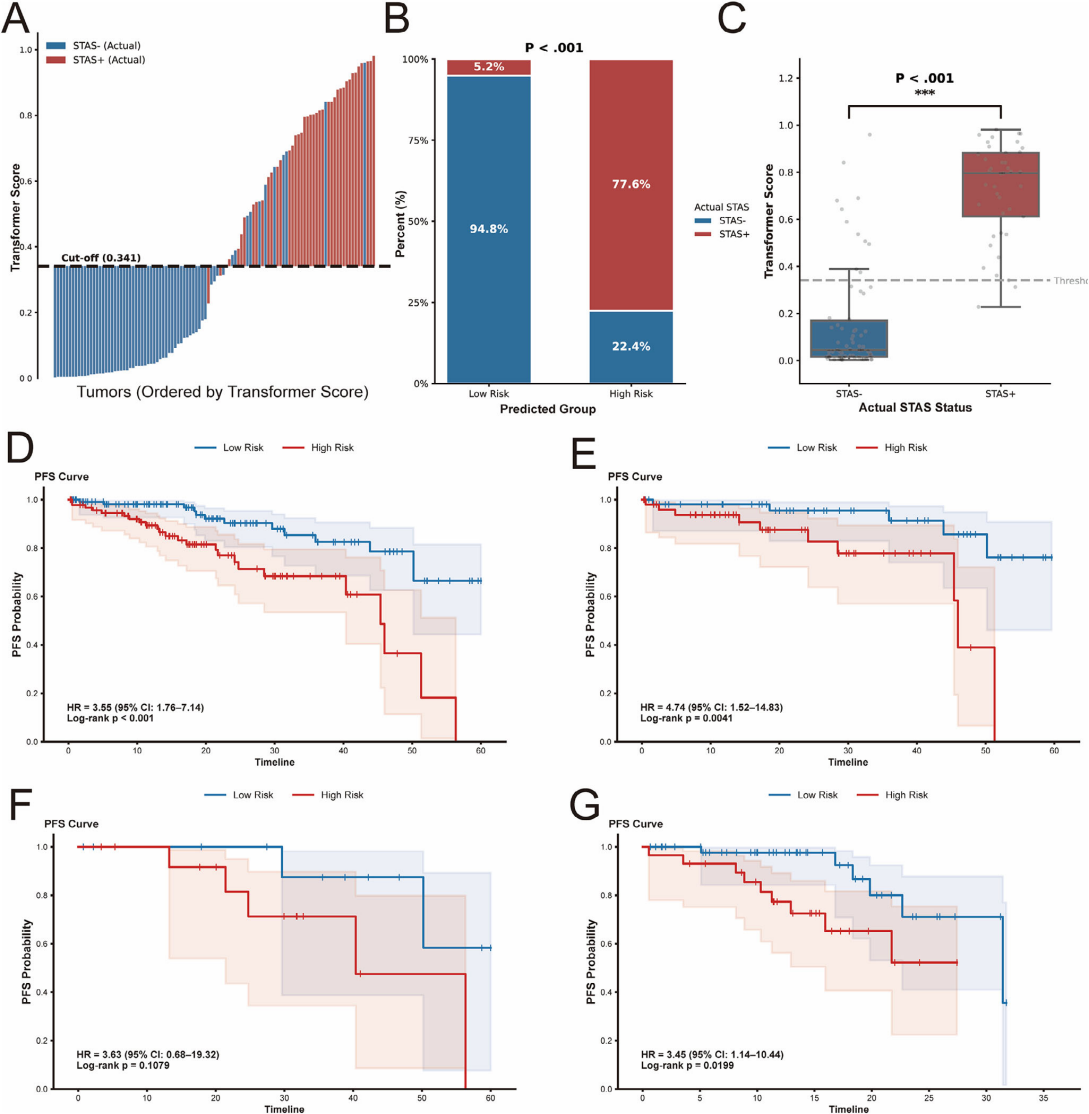
Model prediction performance and prognostic stratification validation. (A) Waterfall plot showing the distribution of Transformer scores for individual tumors; (B) Stacked bar chart illustrating the proportions of pathologically confirmed STAS+ and STAS‐ tumors stratified by the model‐predicted risk groups (Low STAS Risk vs. High STAS Risk). (C) Boxplot comparing the predicted Transformer scores between actual STAS‐ and STAS+ tumor groups; (D–G) patient‐level Kaplan–Meier PFS curves stratified by Transformer‐derived STAS risk score (high‐risk vs. low‐risk, dichotomized at the training‐cohort Youden‐index‐derived threshold). (D) Pooled cohort (*n* = 212 patients); (E) Training cohort (*n* = 107 patients); (F) Internal validation cohort (*n* = 27 patients; exploratory only, statistically underpowered); (G) External test cohort (*n* = 78 patients; principal independent validation cohort). For patients with multiple tumors, the highest tumor‐level risk score was used for patient‐level survival analysis. Hazard ratios (HR) with 95% confidence intervals and log‐rank *P*‐values are displayed within each panel.

In the pooled patient‐level cohort (*n* = 212), the continuous Transformer‐derived STAS risk score remained associated with PFS after adjustment for T stage, N stage, and maximum tumor diameter (HR = 4.24, 95% CI: 1.47–12.25, *P* = 0.008; Figure 5A). The wide confidence interval and the 37 observed progression events (approximately 9.25 events per modeled variable) indicate that the magnitude of this association may be unstable and requires confirmation in larger cohorts. Exploratory subgroup associations were directionally consistent, and no interaction term reached statistical significance (all *P* for interaction > 0.05; Figure 5B). The proportional hazards assumption was not rejected (global Schoenfeld *P* = 0.616). Harrell's C‐index was 0.674 (95% CI: 0.597–0.751).

**FIGURE 5:**
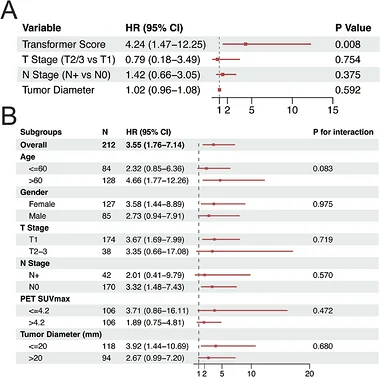
(A) Multivariable Cox proportional hazards regression analysis for progression‐free survival in the pooled cohort. (B) Exploratory subgroup analysis of the association between the Transformer‐defined risk group and progression‐free survival. Hazard ratios with 95% confidence intervals are shown for each subgroup, and *P* values represent tests for interaction.

### 3.6 Exploratory Radiogenomic Analysis

Transcriptomic analysis across the TCGA and GEO cohorts provided exploratory transcriptomic context for the imaging‐defined risk phenotype. Integrating the exploratory GSEA results from both cohorts identified four pathways with concordant directional enrichment (Figure 6A,B). Given the very small TCGA sample (*n* = 14) and the lack of pathological STAS labels in GEO, these findings are hypothesis‐generating and should be interpreted cautiously. Representative leading‐edge genes within each pathway are visualized by heatmap in Figure 6C; the apparent cross‐cohort concordance of individual gene expression patterns (e.g., PCDH12, PCDH19, SYT8, UCP2, FABP4) is noted as hypothesis‐generating rather than mechanistically established [23, 24, 25]. In the TCGA‐LUAD cohort (*n* = 14) and GEO‐NSCLC cohort (*n* = 68), GSEA identified FDR‐significant enrichment of pathways related to cell adhesion suppression, vesicle‐mediated secretion, complement system activation, and fatty acid/uncoupling protein‐associated thermogenesis in the imaging‐defined high‐risk group (Figure 6D,E; all q < 0.05). These results suggest that the imaging‐defined high‐risk phenotype is associated with transcriptional programs related to cell adhesion loss, vesicle trafficking, complement activity, and metabolic remodeling.

**FIGURE 6:**
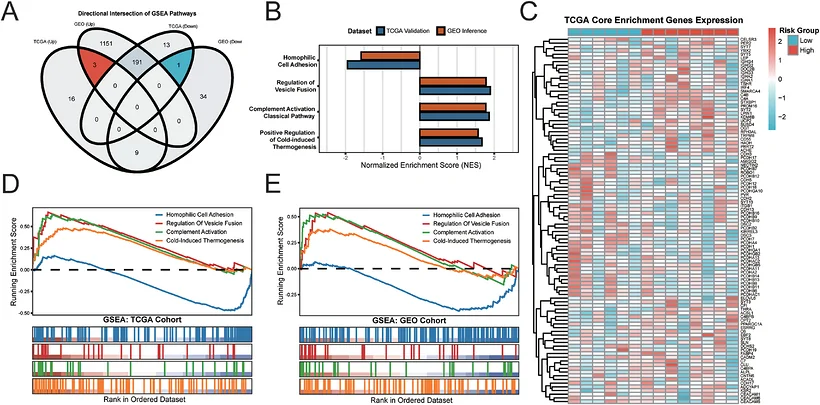
Exploratory radiogenomic analysis of the imaging‐derived STAS risk score. (A) Venn diagram of candidate pathways with concordant directional enrichment across TCGA‐LUAD and GEO‐NSCLC. (B) Descriptive comparison of normalized enrichment scores (NES) for the candidate pathways in TCGA‐LUAD (*n* = 14) and GEO‐NSCLC (*n* = 68). (C) Heatmap of representative leading‐edge genes. (D,E) Representative GSEA plots. Because of the small TCGA cohort and the absence of pathological STAS labels in GEO, these analyses are hypothesis‐generating and not confirmatory molecular validation.

## 4 Discussion

Preoperative prediction of STAS has become a focal point in thoracic oncology, as STAS status may influence the choice between lobectomy and sublobar resection, particularly in patients being considered for parenchyma‐sparing surgery [26]. This multicenter study presents a Transformer‐based multimodal deep learning framework that systematically integrates eight parallel 2.5D PET and CT input streams through a multi‐head self‐attention mechanism for preoperative STAS prediction in resectable LUAD. First, the cross‐modal fusion architecture is designed to capture global inter‐stream dependencies that may be less readily modeled by single‐modality or simple concatenation‐based approaches; second, the continuous model output functions as a prognostically informative risk score that stratifies patients by PFS independently of standard clinicopathological variables; and third, exploratory cross‐cohort transcriptomic analysis provides preliminary biological context linking the imaging phenotype to candidate molecular pathways, generating hypotheses for future mechanistic investigation.

Several CT‐based deep learning approaches have demonstrated promising STAS prediction performance [27, 28, 29]. Wang et al. [27] integrated intratumoral and peritumoral radiomic features with deep learning in a two‐center study (AUC = 0.817 in the validation set), demonstrating the complementary value of peritumoral information; however, their feature concatenation fusion strategy, which aggregates modalities independently rather than modeling cross‐modal interactions, may limit the capture of inter‐regional dependencies. Liu et al. [28] employed an automatic segmentation‐based deep learning model (AUC = 0.88 in the training set), which offers a workflow advantage over manual segmentation but may introduce systematic segmentation errors in morphologically ambiguous lesions. Ma et al. applied a Swin Transformer to CT images for STAS prediction [29], achieving AUC values of 0.869 and 0.837 in the training and validation sets, respectively, supporting the potential of Transformer‐based architectures for STAS prediction. Crucially, these approaches relied exclusively on CT imaging, whereas the present model additionally incorporated PET‐derived metabolic information. In our attention analysis, PET_MSI and CT_MCI showed relatively higher mean attention weights, suggesting that metabolic and morphological information may contribute complementarily within this model. The incremental value of PET integration—beyond the CT‐only approaches—is supported by our ablation results showing AUC improvement in the external test cohort when PET streams are included versus CT‐only Transformer fusion. Nevertheless, attention weights should be interpreted as model‐specific indicators of relative feature utilization rather than direct measures of biological importance.

The dual‐cohort GSEA analysis provides exploratory molecular context for the captured imaging features. In the imaging‐defined high‐risk group, four candidate pathways showed FDR‐significant concordant enrichment across cohorts. Leading‐edge genes PCDH12 and PCDH19 exhibited reduced expression within the Homophilic Cell Adhesion pathway; protocadherin silencing has been associated with increased tumor cell motility in prior experimental studies [23], though whether this contributes to STAS‐type dissemination remains untested. SYT8‐associated Vesicle Fusion enrichment is noted in the context of prior reports linking synaptotagmin dysregulation to tumor secretory phenotypes [24]. Significant C4BPB upregulation within the Complement Activation pathway is consistent with complement regulatory roles described in independent experimental work [30, 31], representing a testable immune‐evasion hypothesis. Finally, FDR‐significant enriched UCP2/FABP4‐associated thermogenesis gene sets are of interest given prior reports linking mitochondrial uncoupling to altered glucose metabolism [25, 32].

These concordant signals may represent candidate transcriptomic correlates of the imaging‐defined high‐risk phenotype; however, they should be regarded as hypothesis‐generating observations. This interpretation is particularly important given the small size of the TCGA subset and the absence of pathological STAS labels in the GEO cohort. Prospective cohorts with matched PET/CT imaging, pathological STAS assessment, and transcriptomic profiling will be required to validate these candidate associations and determine whether they are reproducibly linked to STAS‐related imaging phenotypes.

Clinically, our survival analysis showed that the model‐defined high‐risk group was associated with shorter PFS. The association remained directionally consistent in a sensitivity analysis excluding patients with less than 6 months of follow‐up, suggesting that the observed survival separation was not solely driven by very early events. The preoperative score may be useful as an adjunctive risk marker rather than a stand‐alone indication for a specific operation. A high score could prompt closer multidisciplinary review of resection extent together with tumor size, location, pulmonary reserve, nodal assessment, and other established factors. Because the cutoff was selected by the Youden index in the training cohort, prospective calibration and decision‐impact studies are required before it can be used to choose lobectomy versus sublobar resection in routine practice [33].

Several limitations affect interpretation and clinical translation. First, the retrospective nature introduces potential selection bias, although we mitigated this by using multicenter cohorts. Second, while the sample size (*n* = 212) is substantial for a PET/CT radiogenomic study, it remains modest for large‐scale Transformer training; the observed training‐to‐external‐test AUC gap (0.945 vs. 0.822) suggests residual overfitting despite the use of multiple strategies to reduce overfitting and information leakage, including transfer learning, data augmentation, weight decay, dropout, early stopping, and out‐of‐fold feature extraction. Larger training datasets may further improve model generalizability. Moreover, because the internal validation cohort was used for both early stopping and selection of the highest‐AUC fold‐specific DenseNet models, its performance estimates may be optimistic and should not be interpreted as independent validation. The external multicenter test cohort therefore provides the primary assessment of generalizability. Third, scanner and acquisition heterogeneity remained across the three centers. Although PET and CT images underwent a common resampling, intensity‐standardization, and discretization pipeline, these steps cannot guarantee removal of all domain shift. Fourth, manual segmentation may introduce contour‐dependent variation even though inter‐observer ICCs were 0.88 for PET and 0.82 for CT in a 30‐tumor subset. Automated or semi‐automated segmentation should therefore be validated prospectively, including its effect on prediction stability and workflow time. Fifth, only 37 progression events were available for the four‐covariate Cox model, yielding approximately 9.25 events per variable and relatively wide hazard‐ratio confidence intervals; prognostic effect sizes should therefore be regarded as preliminary.

Sixth, the radiogenomic analysis was exploratory: only 14 TCGA‐LUAD cases had matched PET/CT and RNA sequencing, while the GEO cohort lacked pathological STAS labels. The resulting pathway and gene‐level signals are vulnerable to sampling variability and should not be treated as mechanistic evidence. Seventh, interpretability analyses have their own limitations. Attention weights were summarized at the cohort level, and attribution stability across individuals, model retraining, resampling, or input perturbation was not formally evaluated. Grad‐CAM depends on the selected target layer and visualizes branch‐level rather than Transformer‐level spatial attribution; SHAP results depend on explainer and reference/background choices. Accordingly, all attention, Grad‐CAM, and SHAP findings are descriptive and hypothesis‐generating. Future work should quantify attribution reproducibility and evaluate whether explanation patterns remain stable in larger independent cohorts.

In conclusion, this multicenter study demonstrates that a Transformer‐based multimodal framework integrating eight parallel 2.5D PET and CT input streams can provide preoperative STAS risk estimates in resectable LUAD, with performance evaluated in an independent external test cohort and prognostic stratification that remained informative after adjustment for standard clinicopathological variables. The continuous score was associated with PFS after adjustment for standard clinicopathological variables, but the limited number of progression events requires cautious interpretation. The attribution and transcriptomic analyses provide descriptive, hypothesis‐generating context. Prospective multicenter studies with larger validation cohorts, automated segmentation integration, and matched transcriptomic profiling are warranted to translate this framework toward clinical decision support in individualized surgical planning for LUAD.

## Author Contributions

X. Y. Z., M. M., and Z. Z. W. contributed equally to this work and share co‐first authorship. X. Y. Z., M. M., and Z. Z. W. conceived and designed the study and contributed to data interpretation. D. L. L., Y. M. W., and X. C. M. participated in data acquisition and analysis. X. Y. Z. drafted the initial manuscript. X. Y. M. and W. F. jointly supervised the study and critically revised the manuscript for important intellectual content.

## Conflicts of Interest

The authors declare no conflicts of interest.

## Supporting information




**Supporting File**: advs78076‐sup‐0001‐SuppMat.docx.

## Data Availability

The datasets analyzed during the current study are not publicly available because of institutional restrictions and patient privacy, but are available from the corresponding author on reasonable request and with permission from the participating institutions.
