# Supplementary material for: Transformer‐Based PET/CT Fusion Enables Preoperative Prediction and Prognostic Stratification of Spread Through Air Spaces in Lung Adenocarcinoma
Source: Adv Sci (Weinh). 2026 Sep 29:e78076. Online ahead of print. doi: 10.1002/advs.78076 (PMC13621198; doi:10.1002/advs.78076)
Supplement: Supplementary file 1 [file ADVS-9999-e78076-s001.docx]

**Supporting information**

***Transformer-Based PET/CT Fusion Enables Preoperative Prediction and Prognostic Stratification of Spread Through Air Spaces in Lung Adenocarcinoma***

Xin-Yu Zhu, Meng Meng, Zhen-Zhen Wang, Deng-Lu Lu, Ya-Min Wei, Xu-Chang Mi, Xing-Yu Mu^*^, Wei Fu^*^

X. Y. Z., Z. Z. W., X. Y. M. and W. F.

Department of Nuclear Medicine, The First Affiliated Hospital of Guilin Medical University, Guilin 541001, China

Email: muxingyu1992@hotmail.com; fuwei19700513@163.com

M. M.

Laboratory Center, Guangxi Key Laboratory of Metabolic Reprogramming and Intelligent Medical Engineering for Chronic Diseases, The Second Affiliated Hospital of Guilin Medical University, Guilin 541199, China

Key Laboratory of Medical Biotechnology and Translational Medicine (Guilin Medical University), Education Department of Guangxi Zhuang Autonomous Region, Guilin 541199, China

D. L. L.

Department of Nuclear Medicine, Liuzhou Worker's Hospital, Liuzhou 545005, China

Y. M. W.

Department of Pathology, The First Affiliated Hospital of Guilin Medical University, Guilin 541001, China

X. C. M.

Department of Nuclear Medicine, Nanxishan Hospital, Guilin 545005, China

| **Table of Contents** |  |
| --- | --- |
| Supplementary Note 1-6 | S2-10 |
| Tables **S1-S4** | S11-14 |
| Figures **S1-S6** | S15-21 |

**Supplementary Note 1 Clinical Model**

To establish a performance baseline, a traditional clinical model was constructed using conventional PET/CT quantitative parameters. Based on clinical relevance and findings from previous literature, three key variables were selected for inclusion: maximum standardized uptake value (SUVmax), mean standardized uptake value (SUVmean), and maximum tumor diameter. These variables represent the standard-of-care metrics used in routine clinical evaluation of tumor metabolic activity and size. A multivariable logistic regression (LR) framework was employed to develop the clinical model. It is important to note that these parameters were incorporated to represent the diagnostic potential of standard quantitative imaging, serving as a reference to evaluate the incremental value of the Transformer-based deep learning framework.

**Supplementary Note 2 Radiomics Workflow and Image Acquisition**

**2.1 Image acquisition and preprocessing**

^18^F-FDG PET/CT scans were obtained from three centers: The First Affiliated Hospital of Guilin Medical University, using a Philips Ingenuity TF PET/CT scanner; Guilin Nanxishan Hospital, using a GE Discovery MI PET/CT scanner and Liuzhou Workers' Hospital, using a Siemens BIOGRAPH mCT S(20) - 3R.

Patients refrained from strenuous activity for at least 24 h and fasted for a minimum of 6 h before radiotracer administration, maintaining blood glucose levels below 11 mmol/L (200 mg/dL). After intravenous injection of ^18^F-FDG (3.7 MBq/kg; pH 5–7; purity > 95%), patients rested quietly in the supine position for approximately 60 min. Immediately before imaging, patients were instructed to void and then consume water to achieve gastric distention. CT scans were acquired via the following parameters: 120 kV, automatic current modulation, 780-mm transaxial field of view, 16×1.2 mm collimation, pitch of 0.8, rotation time of 0.5 s, slice thickness of 3 mm, and a 512×512 matrix. Subsequently, PET images were acquired in three-dimensional (3D) mode with an acquisition time of 1.5 min per bed position (zoom factor of 1.0, matrix size of 200×200). PET image reconstruction utilized the ordered subset expectation maximization algorithm (2 iterations, 21 subsets) with a Gaussian post-reconstruction filter of 5-mm full width at half maximum.

Image intensities were converted to standardized uptake values (SUVs) normalized to body weight. All PET images were resampled to isotropic resolution (3 × 3 × 3 mm^3^) using linear interpolation and underwent intensity discretization with a fixed bin width of 0.31 SUV (192 gray levels between 0–60 SUV). For CT images we used lung window (set in a specific range of CT values from -1000 to 300 Hounsfield Units (HU)) with a fixed bin width of 25HU and resampled to a voxel size of 1 × 1 × 1 mm³. These modality-specific preprocessing procedures were applied consistently across the three clinical centers before feature extraction.

**2.2 Traditional Radiomics Models**

Radiomic features were extracted in PyRadiomics (<http://www.radiomics.io/pyradiomics.html>) according to IBSI guidelines[22]. A total of 4032 features were extracted from PET (2016 features) and CT (2016 features) images:792 firstorder features, 28 shape features, 968 gray-level co-occurrence matrix (GLCM) features, 704 gray-level size zone matrix (GLSZM) features, 704 gray-level run-length matrix (GLRLM) features, 220 neighboring gray-tone difference matrix (NGTDM) features, 616 gray-level dependence matrix (GLDM) features.

Radiomic features were standardized using Z-score normalization, with mean and standard deviation values calculated from the training cohort and then applied unchanged to the internal validation and external test cohorts.. Feature selection was performed exclusively within the training cohort using the minimum redundancy maximum relevance (mRMR) algorithm. The 20 highest-ranked features were retained for subsequent LASSO regression. Predictive features were finalized via Least absolute shrinkage and selection operator (LASSO) regression with 10-fold cross-validation to identify the most radiologically meaningful predictors. Eight supervised machine learning classifiers were trained on the selected features: Logistic Regression, support vector machine (SVM), KNN, XGBoost, LightGBM, Random Forest, ExtraTrees, and multilayer perceptron (MLP). Subsequently, we adopted 5-fold cross-validation to evaluate the robustness of model performance. We evaluated the models using ROC curves and DCA curves to analyze their performance and the impact of classification thresholds.

**2.3: 2.5D image segmentation**

Multi-Planar Imaging (MPI) focused on the largest axial cross-section of the tumor and generated three variants: a strict intratumoral slice (ROI-only), a full maximal cross-sectional slice within the tumor-bounding cuboid (ROI-all), and a peritumoral-expanded slice obtained by extending the contour outward by three voxels (enlarge-3), enabling simultaneous assessment of intra- and peri-tumoral features. Multi-Slice Imaging (MSI) preserved limited volumetric continuity by extracting three consecutive axial slices (−2, 0, +2) centered on the maximal cross-section to incorporate subtle superior–inferior variations. Multicenter Imaging (MCI) sampled orthogonal central planes (axial, sagittal, and coronal) passing through the tumor centroid and extracted three core layers per plane to ensure structural consistency across anatomical axes. Multi-Angle Imaging (MAI) further introduced orientation diversity by generating 45° oblique views across the principal planes and integrating three-layer edge-focused slices to encode boundary-related spatial characteristics.

**Supplementary Note 3 2.5D Models**

3.1 **DenseNet161 architecture**

The DenseNet161 architecture is characterized by its dense connectivity pattern, where each layer receives additional inputs from all preceding layers and passes on its own feature maps to all subsequent layers. The model begins with an initial convolutional stage consisting of a 7×7 convolutional layer (conv1) with stride 2, padding 3, and 96 output channels, followed by Batch Normalization and ReLU activation. This is succeeded by a 3×3 max pooling layer with stride 2 for initial downsampling. The core of the architecture is composed of four sequential Dense Blocks interspersed with Transition Layers. DenseNet161 utilizes a growth rate (k) of 48 and a bottleneck structure within each dense layer to improve computational efficiency. Dense Block 1 contains 6 bottleneck layers. Each layer sequentially applies Batch Normalization, ReLU, a 1×1convolution (producing 192 channels, or 4k), followed by Batch Normalization, ReLU, and a 3×3 convolution producing 48 channels, which are then concatenated with the input feature maps. Transition Layer 1 follows, comprising a 1×1 convolution that compresses the channel dimension by a factor of θ=0.5, followed by a 2×2 average pooling layer with stride 2. Dense Block 2 consists of 12 bottleneck layers, followed by Transition Layer 2 which further reduces spatial resolution and channel depth. Dense Block 3 is the deepest section, containing 36 bottleneck layers, followed by Transition Layer 3. Dense Block 4 concludes the feature extraction stage with 24 bottleneck layers. The network terminates with a classification head: a final Batch Normalization and ReLU sequence, followed by an adaptive global average pooling layer that collapses the spatial dimensions into 1 \times 1 feature maps. For regularization, a dropout layer with a rate of 0.2 was applied, leading into the final fully-connected (fc) layer. This layer maps the 2208 input features (the final concatenated channel depth of DenseNet161) to 2 output features for binary classification. This dense connectivity design significantly enhances feature reuse, mitigates the vanishing-gradient problem, and strengthens feature propagation throughout the deep network.

**3.2 Parameters Configuration**

params = dict( normalize_method='imagenet',

batch_size=32,

epochs=100,

init_lr=0.01,

dropout=0.2,

weight_decay=1e-4,

optimizer='sgd',

in_channels=3,

pretrained=True)

**3.3 Out-of-Fold Feature Extraction**

To generate training-cohort features for downstream Transformer fusion while minimizing information leakage, a stratified five-fold out-of-fold (OOF) strategy was used. The training cohort was divided into five folds at the tumor level, with the proportions of STAS-positive and STAS-negative tumors maintained as closely as possible across folds. A fixed random seed of 42 was used for reproducible fold assignment.

In each iteration, four folds were used to train the corresponding DenseNet161 branch, whereas the remaining fold was excluded from model training and used only for feature extraction. This procedure was repeated five times so that every tumor in the training cohort received a feature representation generated by a model that had not been trained using that tumor or its pathological label. Because each patient in the training cohort contributed only one tumor, tumor-level fold assignment was equivalent to patient-level separation and therefore did not introduce patient overlap between the training and held-out subsets.

For each of the eight 2.5D streams, the five-fold-specific DenseNet161 models were subsequently evaluated on the internal validation cohort. The model achieving the highest AUC for STAS classification was selected as the branch-specific inference model and its parameters were thereafter fixed. This selected model was used to extract the corresponding 2,208-dimensional feature vectors from both the internal validation and external test cohorts. No averaging or ensembling of the five-fold-specific models was performed, and the DenseNet161 backbone was not retrained on the full training cohort after completion of the five-fold OOF procedure.

For tumors in the training cohort, only the feature vector generated by the corresponding held-out OOF model was retained for downstream Transformer fusion. Thus, training-cohort Transformer inputs were derived from OOF feature extraction, whereas internal-validation and external-test inputs were generated using the selected branch-specific inference models. The external test cohort was not involved in fold assignment, DenseNet training, early stopping, branch-model selection, or hyperparameter optimization.

**3.4 Grad-CAM heatmaps**

Grad-CAM was applied separately to each independently trained DenseNet161 branch to visualize the image regions contributing to branch-level STAS prediction. The final convolutional block (denseblock.denselayer24.conv2) was used as the target layer. For Grad-CAM visualization, the branch-specific DenseNet161 model selected according to the highest internal-validation AUC, as described in Supplementary Note 3.3, was used. The model was set to evaluation mode, and its parameters were kept fixed and were not updated during Grad-CAM computation.

Each 2.5D input consisted of three image layers stacked along the channel dimension. The same preprocessing used during model inference was applied before visualization, including resizing to 256 x 256 pixels and ImageNet normalization consistent with branch-model input preprocessing. The input tensor was converted from [H, W, C] to [1, C, H, W] and transferred to the computation device. For each case, gradients were calculated with respect to the STAS-positive output logit of the corresponding DenseNet161 branch. Gradients at the target convolutional layer were globally averaged over spatial dimensions to obtain channel-wise weights. Weighted activation maps were summed, rectified using ReLU, normalized to [0,1], and upsampled to the input resolution using bilinear interpolation.

Grad-CAM visualizations were generated independently for all eight branches. The cases shown in Figures 3C, D and Figures S4A, B were selected from the internal validation cohort using a fixed random seed of 42. One STAS-positive case and one STAS-negative case were displayed. These cases were selected for qualitative illustration and were not used to quantify localization accuracy, attribution stability, or biological mechanism. Because the Grad-CAM maps were generated from the individual DenseNet161 branch outputs, they represent branch-level spatial attribution and do not directly visualize the final Transformer fusion decision.

**Supplementary Note 4 Details of Transformer Model**

**4.1: Transformer architecture**

Prior to Transformer input, the 2,208-dimensional deep feature vectors were standardized using z-score normalization. For each feature dimension, the mean and standard deviation were estimated exclusively from the training cohort and subsequently fixed and applied unchanged to the internal validation and external test cohorts. No normalization parameters were estimated from either validation cohort. The input feature tokens with dimensions of 32×8×2208 (batch × sequence × features) were processed without positional encodings, as the eight modality/view representations were treated as an unordered set of feature tokens rather than a spatially or temporally ordered sequence. Each 2208-dimensional token was first projected onto a 512-dimensional latent space. A learnable class token ([CLS]) was appended to the sequence to aggregate global representations. The Transformer encoder consisted of two layers with 16 attention heads. For the final binary prediction, the [CLS] token state was passed through a two-node output layer. The model was optimized using the cross-entropy loss with the Adam optimizer. To prevent overfitting, layer normalization and a weight decay of 1e-4 were applied. Training was conducted for 60 epochs with a batch size of 32 and an initial learning rate of 0.01, which was adjusted using a Cosine Annealing scheduler. Hyperparameters were selected empirically during model development based on training convergence and performance in the internal validation cohort. No exhaustive grid search, random search, or automated hyperparameter optimization was performed. Once the final configuration was determined, all hyperparameters were fixed before evaluation in the independent external test cohort, which was not used for hyperparameter tuning or model selection.

**4.2: SHapley Additive exPlanations**

Interpretability analyses were performed after model training with all model parameters fixed. Attention weights were summarized across the training cohort as mean ± SEM.

For complementary post hoc attribution, SHAP analysis was implemented using KernelExplainer. Rather than attributing the 17,664 individual deep features separately, the eight PET/CT streams (CT_MAI, CT_MCI, CT_MPI, CT_MSI, PET_MAI, PET_MCI, PET_MPI, and PET_MSI) were treated as eight grouped features, with each group comprising its corresponding 2,208-dimensional DenseNet161 feature vector. For each explained case, Kernel SHAP operated on an eight-element binary inclusion mask. For consistency across interpretability analyses, SHAP was performed on the same representative STAS-positive and STAS-negative cases from the internal validation cohort as those used for the corresponding Grad-CAM visualizations.When a stream was included, its complete patient-specific 2,208-dimensional feature vector was retained; when excluded, the entire stream was replaced by the feature-wise mean reference vector derived from standardized training-cohort feature matrix. The all-reference input therefore corresponded to an eight-stream input in which every stream was replaced by its respective mean reference representation.

The explained model output was the pre-softmax logit for the STAS-positive class rather than the post-softmax probability. The SHAP expected value represented the STAS-positive logit produced by the all-reference input, and the eight resulting SHAP values represented additive stream-level deviations from this base value. Positive SHAP values therefore indicated that a given stream increased the STAS-positive logit relative to the reference input, whereas negative values indicated a decrease. Stream-level contributions were obtained directly through grouped masking and were not generated by post hoc aggregation of individual feature-level SHAP values.

Kernel SHAP estimation was performed using 1000 samples for each explained case, with the random seed fixed at 42. Waterfall and force plots were generated from the same SHAP values and corresponding expected value. Because SHAP estimates are dependent on the selected reference representation and model structure, these outputs were used solely for descriptive interpretation of individual model behavior and were not regarded as causal or mechanistic measures of feature importance.

**Supplementary Note 5 Results of Radiomics and Clinical models**

Univariable t tests identified 2,315 candidate features. Redundancy filtering based on pairwise Pearson correlation (|r| > 0.90) retained one feature from each highly correlated pair, leaving 272 features for subsequent selection. Using mRMR and LASSO regression, we selected 7 features to build the radiomic model (Figure S2A). The MLP radiomics model achieved an AUC of 0.770 (95% CI, 0.666 - 0.873) in the external test cohort, with an accuracy of 0.767, precision of 0.640, and F1 score of 0.615. Figure S2C shows the ROC curves across the training, internal validation, and external test cohorts. The clinical model showed lower discrimination in the external test cohort (AUC, 0.736; 95% CI, 0.620 - 0.851; Figure S2D).

**Supplementary Note 6** **Ablation analysis**

To compare the proposed Transformer-based fusion strategy with alternative fusion approaches, we conducted an ablation analysis, we conducted a systematic ablation study comparing: (1) Transformer fusion (proposed); (2) Early fusion (feature concatenation followed by MLP); (3) Late fusion (LR stacking of individual branch predictions) and (4) Transformer_MCI (best PET/CT single branch transformer fusion). The Transformer fusion model achieved the highest AUC in the external test cohort (0.822 (95% CI: 0.725–0.920)), outperforming the next-best strategy (Late fusion, AUC =0.746, 95% CI: 0.627–0.866), demonstrating that the Transformer fusion model with self-attention mechanism achieved the highest AUC among the evaluated fusion strategies in the external test cohort.

| **Table S1. Baseline clinicopathological and PET/CT characteristics of tumors in the training, internal validation, and external test cohorts** | | | | | |
| --- | --- | --- | --- | --- | --- |
| Characteristics | Total (n=219) | Training (n=107) | Internal validation (n=27) | External test (n=85) | P value^*^ |
| Age (years) | 60.99±8.70 | 60.31±9.31 | 61.41±11.16 | 61.72±6.91 | 0.109 |
| Sex |  |  |  |  | 0.123 |
| Female | 134 (61.2) | 61(57.1) | 14(51.9) | 59(69.4) |  |
| Male | 85(38.8) | 46(42.9) | 13(48.1) | 26(30.6) |  |
| Maximum tumor diameter (mm) | 21.67±10.10 | 23.15±10.93 | 21.19±9.49 | 20.02±8.96 | 0.097 |
| SUV_max_ | 5.61±4.87 | 5.45±5.02 | 6.61±5.60 | 5.51±4.87 | 0.526 |
| SUV_mean_ | 3.42±2.97 | 3.31±3.08 | 4.01±3.47 | 3.36±2.67 | 0.539 |
| SUV_peak_ | 3.98±3.38 | 3.72±2.59 | 4.02±3.77 | 4.67±3.50 | 0.434 |
| 40%_TLG | 25.32±76.30 | 33.63±100.09 | 23.67±46.31 | 15.51±40.82 | 1.000 |
| T |  |  |  |  | 0.308 |
| T1 | 181(82.6) | 83(77.6) | 23(85.2) | 75(88.3) |  |
| T2 | 33(15.1) | 20(18.7) | 4(14.8) | 9(10.5) |  |
| T3 | 5(2.3) | 4(3.7) | 0 | 1(1.2) |  |
| N |  |  |  |  | 0.330 |
| N0 | 176(80.4) | 84(78.5) | 21(77.78) | 71(83.5) |  |
| N1 | 27(12.3) | 16(15.0) | 5(18.52) | 6(7.0) |  |
| N2 | 16(7.3) | 7(6.5) | 1(3.70) | 8(9.5) |  |
| M |  |  |  |  | 1 |
| M0 | 219(100.0) | 107(100.0) | 27(100.0) | 85(100.0) |  |
| Data are presented as n (%) for categorical variables and mean ± standard deviation (SD) for continuous variables. | | | | | |
| ^*^P values were calculated using one-way ANOVA for continuous variables and the chi-square test or Fisher's exact test for categorical variables, as appropriate. | | | | | |

| **Table S2. Comprehensive comparison of the performance of the radiomic modules** | | | | | | | | | | |
| --- | --- | --- | --- | --- | --- | --- | --- | --- | --- | --- |
| model_name | Accuracy | AUC | 95% CI | Sensitivity | Specificity | PPV | NPV | F1 | Threshold | Cohort |
| LR | 0.701 | 0.811 | 0.7318 - 0.8898 | 0.976 | 0.53 | 0.563 | 0.972 | 0.714 | 0.215 | Training |
| LR | 0.605 | 0.712 | 0.6051 - 0.8199 | 0.963 | 0.441 | 0.441 | 0.963 | 0.605 | 0.283 | External test |
| LR | 0.741 | 0.778 | 0.5962 - 0.9593 | 0.778 | 0.722 | 0.583 | 0.867 | 0.667 | 0.435 | Internal validation |
| SVM | 0.85 | 0.911 | 0.8545 - 0.9666 | 0.805 | 0.879 | 0.805 | 0.879 | 0.805 | 0.487 | Training |
| SVM | 0.651 | 0.704 | 0.5900 - 0.8186 | 0.704 | 0.627 | 0.463 | 0.822 | 0.559 | 0.404 | External test |
| SVM | 0.852 | 0.772 | 0.5377 - 1.0000 | 0.667 | 0.944 | 0.857 | 0.85 | 0.75 | 0.706 | Internal validation |
| KNN | 0.832 | 0.882 | 0.8203 - 0.9436 | 0.829 | 0.833 | 0.756 | 0.887 | 0.791 | 0.6 | Training |
| KNN | 0.477 | 0.584 | 0.4649 - 0.7027 | 0.889 | 0.288 | 0.364 | 0.85 | 0.516 | 0.4 | External test |
| KNN | 0.630 | 0.673 | 0.4593 - 0.8864 | 0.889 | 0.5 | 0.471 | 0.9 | 0.615 | 0.4 | Internal validation |
| RandomForest | 0.860 | 0.920 | 0.8714 - 0.9693 | 0.902 | 0.833 | 0.771 | 0.932 | 0.831 | 0.441 | Training |
| RandomForest | 0.640 | 0.761 | 0.6523 - 0.8699 | 0.889 | 0.525 | 0.462 | 0.912 | 0.608 | 0.314 | External test |
| RandomForest | 0.815 | 0.784 | 0.5408 - 1.0000 | 0.778 | 0.833 | 0.700 | 0.882 | 0.737 | 0.53 | Internal validation |
| ExtraTrees | 0.776 | 0.89 | 0.8312 - 0.9485 | 0.927 | 0.682 | 0.644 | 0.937 | 0.760 | 0.379 | Training |
| ExtraTrees | 0.709 | 0.757 | 0.6489 - 0.8646 | 0.704 | 0.712 | 0.528 | 0.84 | 0.603 | 0.429 | External test |
| ExtraTrees | 0.815 | 0.796 | 0.6089 - 0.9837 | 0.556 | 0.944 | 0.833 | 0.81 | 0.667 | 0.504 | Internal validation |
| XGBoost | 0.972 | 0.996 | 0.9896 - 1.0000 | 0.951 | 0.985 | 0.975 | 0.97 | 0.963 | 0.558 | Training |
| XGBoost | 0.535 | 0.639 | 0.5194 - 0.7587 | 0.926 | 0.356 | 0.397 | 0.913 | 0.556 | 0.23 | External test |
| XGBoost | 0.630 | 0.691 | 0.4662 - 0.9165 | 0.889 | 0.5 | 0.471 | 0.9 | 0.615 | 0.323 | Internal validation |
| LightGBM | 0.850 | 0.896 | 0.8368 - 0.9552 | 0.805 | 0.879 | 0.805 | 0.879 | 0.805 | 0.442 | Training |
| LightGBM | 0.686 | 0.652 | 0.5306 - 0.7738 | 0.519 | 0.763 | 0.5 | 0.776 | 0.509 | 0.442 | External test |
| LightGBM | 0.741 | 0.67 | 0.4197 - 0.9198 | 0.778 | 0.722 | 0.583 | 0.867 | 0.667 | 0.456 | Internal validation |
| MLP | 0.748 | 0.839 | 0.7658 - 0.9127 | 0.854 | 0.682 | 0.625 | 0.882 | 0.722 | 0.336 | Training |
| MLP | 0.767 | 0.77 | 0.6661 - 0.8731 | 0.593 | 0.847 | 0.64 | 0.82 | 0.615 | 0.488 | External test |
| MLP | 0.778 | 0.796 | 0.6177 - 0.9749 | 0.667 | 0.833 | 0.667 | 0.833 | 0.667 | 0.504 | Internal validation |

| **Table S3. Comprehensive comparison of the performance of the eight Deep learning modules** | | | | | | | | | |
| --- | --- | --- | --- | --- | --- | --- | --- | --- | --- |
| **Signature** | **Accuracy** | **AUC** | **95% CI** | **Sensitivity** | **Specificity** | **PPV** | **NPV** | **F1** | **Threshold** |
| The diagnostic metrics for the eight DL models in Training cohort. | | | | | | | | | |
| CT_MAI | 0.701 | 0.785 | 0.6943 - 0.8748 | 0.854 | 0.606 | 0.574 | 0.87 | 0.686 | 0.36 |
| CT_MCI | 0.776 | 0.815 | 0.7350 - 0.8955 | 0.585 | 0.894 | 0.774 | 0.776 | 0.667 | 0.505 |
| CT_MPI | 0.766 | 0.811 | 0.7260 - 0.8963 | 0.683 | 0.818 | 0.7 | 0.806 | 0.691 | 0.554 |
| CT_MSI | 0.776 | 0.83 | 0.7535 - 0.9073 | 0.854 | 0.727 | 0.66 | 0.889 | 0.745 | 0.421 |
| PET_MAI | 0.701 | 0.684 | 0.5757 - 0.7931 | 0.537 | 0.803 | 0.629 | 0.736 | 0.579 | 0.413 |
| PET_MCI | 0.71 | 0.785 | 0.6991 - 0.8700 | 0.854 | 0.621 | 0.583 | 0.872 | 0.693 | 0.332 |
| PET_MPI | 0.626 | 0.65 | 0.5418 - 0.7576 | 0.707 | 0.576 | 0.509 | 0.76 | 0.592 | 0.366 |
| PET_MSI | 0.72 | 0.747 | 0.6500 - 0.8437 | 0.756 | 0.697 | 0.608 | 0.821 | 0.674 | 0.323 |
| The diagnostic metrics for the eight DL models in internal validation cohort. | | | | | | | | | |
| CT_MAI | 0.731 | 0.627 | 0.3776 - 0.8773 | 0.556 | 0.824 | 0.625 | 0.778 | 0.588 | 0.824 |
| CT_MCI | 0.654 | 0.778 | 0.5932 - 0.9623 | 1 | 0.471 | 0.5 | 1 | 0.667 | 0.418 |
| CT_MPI | 0.808 | 0.856 | 0.7128 - 0.9996 | 1 | 0.706 | 0.643 | 1 | 0.783 | 0.396 |
| CT_MSI | 0.692 | 0.68 | 0.4709 - 0.8886 | 0.778 | 0.647 | 0.538 | 0.846 | 0.636 | 0.27 |
| PET_MAI | 0.808 | 0.758 | 0.5598 - 0.9566 | 1 | 0.706 | 0.643 | 1 | 0.783 | 0.437 |
| PET_MCI | 0.846 | 0.85 | 0.6948 - 1.0000 | 0.889 | 0.824 | 0.727 | 0.933 | 0.8 | 0.503 |
| PET_MPI | 0.731 | 0.536 | 0.2711 - 0.8008 | 0.333 | 0.941 | 0.75 | 0.727 | 0.462 | 0.413 |
| PET_MSI | 0.769 | 0.634 | 0.3525 - 0.9155 | 0.556 | 0.882 | 0.714 | 0.789 | 0.625 | 0.502 |
| The diagnostic metrics for the eight DL models in external test cohort. | | | | | | | | | |
| CT_MAI | 0.776 | 0.725 | 0.6082 - 0.8427 | 0.593 | 0.862 | 0.667 | 0.82 | 0.627 | 0.479 |
| CT_MCI | 0.776 | 0.663 | 0.5251 - 0.8006 | 0.481 | 0.914 | 0.722 | 0.791 | 0.578 | 0.549 |
| CT_MPI | 0.741 | 0.741 | 0.6187 - 0.8628 | 0.63 | 0.793 | 0.586 | 0.821 | 0.607 | 0.327 |
| CT_MSI | 0.682 | 0.743 | 0.6273 - 0.8580 | 0.741 | 0.655 | 0.5 | 0.844 | 0.597 | 0.116 |
| PET_MAI | 0.576 | 0.644 | 0.5189 - 0.7685 | 0.815 | 0.466 | 0.415 | 0.844 | 0.55 | 0.348 |
| PET_MCI | 0.541 | 0.622 | 0.4904 - 0.7536 | 0.852 | 0.397 | 0.397 | 0.852 | 0.541 | 0.382 |
| PET_MPI | 0.671 | 0.584 | 0.4542 - 0.7131 | 0.519 | 0.741 | 0.483 | 0.768 | 0.5 | 0.381 |
| PET_MSI | 0.753 | 0.688 | 0.5634 - 0.8134 | 0.444 | 0.897 | 0.667 | 0.776 | 0.533 | 0.478 |
| AUC, Area Under the Curve; CI, Confidence Interval; PPV, Positive Predictive Value; NPV, Negative Predictive Value. | | | | | | | | | |

| **Table S4. Ablation analysis** | | | | | | | | | | |
| --- | --- | --- | --- | --- | --- | --- | --- | --- | --- | --- |
| model_name | Accuracy | AUC | 95% CI | Sensitivity | Specificity | PPV | NPV | F1 | Threshold | Cohort |
| Early Fusion | 0.869 | 0.918 | 0.8623 - 0.9728 | 0.854 | 0.879 | 0.814 | 0.906 | 0.833 | 0.426 | Training |
| Early Fusion | 0.718 | 0.706 | 0.5808 - 0.8317 | 0.741 | 0.707 | 0.541 | 0.854 | 0.625 | 0.394 | External test |
| Early Fusion | 0.692 | 0.588 | 0.3453 - 0.8311 | 0.667 | 0.706 | 0.545 | 0.800 | 0.600 | 0.402 | Internal validation |
| Late Fusion | 0.953 | 0.973 | 0.9398 - 1.0000 | 0.902 | 0.985 | 0.974 | 0.942 | 0.937 | 0.553 | Training |
| Late Fusion | 0.741 | 0.746 | 0.6272 - 0.8657 | 0.704 | 0.759 | 0.576 | 0.846 | 0.633 | 0.275 | External test |
| Late Fusion | 0.808 | 0.837 | 0.6627 - 1.0000 | 0.778 | 0.824 | 0.700 | 0.875 | 0.737 | 0.492 | Internal validation |
| Transformer_MCI | 0.822 | 0.859 | 0.7876 - 0.9297 | 0.854 | 0.803 | 0.729 | 0.898 | 0.787 | 0.613 | Training |
| Transformer_MCI | 0.812 | 0.762 | 0.6408 - 0.8834 | 0.593 | 0.914 | 0.762 | 0.828 | 0.667 | 0.482 | External test |
| Transformer_MCI | 0.852 | 0.867 | 0.7294 - 1.0000 | 0.889 | 0.833 | 0.727 | 0.937 | 0.800 | 0.511 | Internal validation |
| Transformer | 0.879 | 0.945 | 0.9023 - 0.9858 | 0.951 | 0.833 | 0.780 | 0.965 | 0.857 | 0.451 | Training |
| Transformer | 0.765 | 0.822 | 0.7247 - 0.9196 | 0.778 | 0.759 | 0.600 | 0.880 | 0.677 | 0.324 | External test |
| Transformer | 0.808 | 0.824 | 0.6600 - 0.9871 | 0.889 | 0.765 | 0.667 | 0.929 | 0.762 | 0.513 | Internal validation |

**Figure S1.** The flowchart illustrates the patient enrollment process for the study

**Figure S2.** Radiomics feature selection and performance evaluation of predictive models. (A) LASSO coefficient profiles of the radiomic features; **(B)** The importance of the 7 final selected radiomics features from CT and PET imaging, ranked by their LASSO coefficients; **(C)** ROC curves for the MLP radiomic model across the training, internal validation, and external test cohorts; **(D)** ROC curves for the LR clinical model across the training, internal validation, and external test cohorts; **(E)** Correlation heatmap between clinical features.

**Figure S3.** (A) ROC curves in the internal validation cohort; (B) decision curve analysis (DCA) in the internal validation cohort; (C) calibration curves in the internal validation cohort; (D) ROC curves in the external test cohort; (E) decision curve analysis (DCA) in the external test cohort; (F) calibration curves in the external test cohort; (G) DeLong test in the training cohort; (H) DeLong test in the internal validation cohort; (I) DeLong test in the external test cohort.

**Figure S4.** (A-B) Grad-CAM visualizations for CT and PET modules; (C) SHAP force plot for a representative STAS-negative case from the internal validation cohort; (D) corresponding grouped stream-level SHAP contributions relative to the training-cohort-derived reference representation.

**Figure S5.** (A-C) waterfall plot, stacked bar chart, and boxplot for the internal validation cohort. (D-E) heatmaps of tumor-level outcomes and multivariable features in the internal validation and external test cohorts, respectively. (F-H) waterfall plot, stacked bar chart, and boxplot for the external test cohort.

**Figure S6.** Prognostic stratification and clinical outcome validation excluding patients with less than 6 months of follow-up. (A) Kaplan-Meier curves for PFS in the pooled population; (B-D) PFS Kaplan-Meier curves for individual cohorts.
